# “We Need to Live off the Land”: An Exploration and Conceptualization of Community-Based Indigenous Food Sovereignty Experiences and Practices

**DOI:** 10.3390/ijerph20054627

**Published:** 2023-03-06

**Authors:** Brianna Poirier, Hannah Tait Neufeld

**Affiliations:** 1 Department of Family Relations and Applied Nutrition, The University of Guelph, Guelph, ON N1G 2W1, Canada; 2 Indigenous Oral Health Unit, The University of Adelaide, Adelaide 5005, Australia; 3 School of Public Health and Health Systems, The University of Waterloo, Waterloo, ON N2L 3G1, Canada

**Keywords:** Indigenous food sovereignty, traditional food, Indigenous Knowledge, sustainability, community based participatory research, Indigenous methodologies, two-eyed seeing

## Abstract

Although Indigenous food systems are sustainable in nature, many of these systems have been forcibly altered among Indigenous communities within Canada, as a consequence of colonization. Indigenous Food Sovereignty (IFS) movements work to counteract the dismantling of Indigenous food systems as well as the negative health impacts of environmental dispossession experienced by Indigenous communities. Through community-based participatory research methodologies and the utilization of Etuaptmumk, or two-eyed seeing, this research project explored community perspectives of IFS in Western Canada. Reflexive thematic analysis of qualitative data collected during a sharing circle held with community members identified the influence of Indigenous Knowledge and community support on three main aspects of Indigenous food sovereignty, including (1) environmental concerns, (2) sustainable practices and (3) a strong relationship with the land and waters. Through the sharing of stories and memories related to traditional foods and current sovereignty projects, community members identified concerns for their local ecosystem as well as a desire to preserve its natural state for generations to come. The strengthening of IFS movements is critical to the overall wellbeing of Indigenous communities within Canada. Support is needed for movements that honour relationships with traditional foods and recognize traditional lands and waters as necessary for healing and sustaining the health of Indigenous communities.

## 1. Introduction

Indigenous Peoples (the term Indigenous Peoples includes both non-status and status First Nation, Inuit and Métis Peoples within Canada [1]) have been deeply connected to the plentiful resources of the land and waters since time immemorial. The Canadian government’s policies aimed at assimilation have undermined Indigenous cultures and suppressed identities, displacing families and communities [2]. Indigenous Peoples continue to actively resist the negative impacts of the historical and present legacies of colonization on the health and wellbeing of communities [3]. Indigenous resistance efforts have focused on the struggle to reclaim Territories and unceded lands that were lost as a result of oppressive government policies aimed at Indigenous assimilation; based on the inhumane assumption that Indigenous Peoples in Canada were inferior and unequal to the settler population [4]. Environmental dispossession resulted from the culminating impacts of government policies, such as the Indian Act, which led to the forcible removal of Indigenous Peoples from their Territories, effectively severing Indigenous Peoples’ relationships with their lands and waters. Richmond and Ross [5] define environmental dispossession as the processes by which Indigenous People’s access to land and resources are decreased; including direct processes such as the physical removal from Territories and indirect processes of colonial and government policies aimed at diminishing Indigenous Knowledge (Indigenous Knowledge (IK) is embedded in a relational understanding of our world, developed through observations, teachings, and revelations that are comprised of visions, dreams, intuition and cellular memory [6]) and access to lands and waters, such as residential schools [5,7]. The consumption of traditional foods has been identified as one of the most direct links between the health of Indigenous Peoples and their Territories. Traditional foods are derived from the local environment, either through the harvesting of wild foods or farming practices [8,9]. These foods and foodways are critical for Indigenous health, social and environmental wellbeing, and play a significant role in the preservation of IK [10,11,12]. Therefore, by stripping Indigenous communities of their sovereignty, processes of environmental dispossession have directly contributed to increased rates of health disparities [3,5,13,14,15]. Loss of Territory is one of the most significant contributors to decreased transfer of IK and the increased prevalence of negative health outcomes across generations of Indigenous Peoples within Canada [16,17].

Indigenous food systems include all food sources available from local resources that are culturally accepted by a community, as well as the sociocultural associations, acquisition techniques, composition and nutritional benefits associated with these foods and practices [13]. Indigenous food systems are complex and cyclical networks, rooted in circumstances that encourage biodiversity and touch the full spectrum of life in ways that modern food systems do not. Additionally, Indigenous food systems are tied to elements of nature and culture that contribute to physical, emotional, mental and spiritual health, providing both healing and protection from illness [8]. Compromised access to Territory has decreased the procurement of traditional foods and increased the reliance on commercially produced foods; this transition has resulted in decreased accessibility of Indigenous food systems [5,10,18].

Previous research has successfully investigated the reality of food (in)security in urban and rural Indigenous communities; this literature focuses on nutrient consumption and typically does not address the impacts of IK and Indigenous food systems on food security [19]. As defined by the Food and Agriculture Organization (FAO), food security occurs when *“all people, at all times, have physical and economic access to sufficient safe and nutritious food that meets their dietary needs and food preferences for an active and healthy life”* [20]. Food sovereignty moves beyond the food security pillars of stability, availability, accessibility and utilization to generate a more holistic understanding of local food contexts and documents the ways in which power relations impact food production, consumption and distribution [10,20,21,22]. In 1996, the term food sovereignty was established by a group of Latin American land-based farmers referred to collectively as La Via Campesina, in protest to the globalization of their local food systems [23]. Food sovereignty is defined as the autonomy or control of a food system and is often used to improve various facets of food security through ecologically sustainable methods [18,24]. Food sovereignty encompasses community well-being, while food security focuses on individualized health and nutritional status; for this reason, food sovereignty efforts improve community self-reliance and support individuals in defining their own food systems [20,25,26]. In response to the effects of colonization on Indigenous Peoples, both Elders and activists across Canada are creating opportunities for the sharing of IK, affirming the significance of Indigenous food systems and the importance of Indigenous Food Sovereignty (IFS) movements [27].

IFS is not only an aspiration for Indigenous communities, but it is a remembered reality that lives on through the intergenerational sharing of IK and food practices [28]. As both a framework and a movement IFS goes beyond food security and sovereignty and aims to reconnect Indigenous Peoples with their food systems through four principles. The first principle is the recognition that food is a sacred gift from the Creator and therefore should not be controlled by colonial influences. Second, IFS is participatory and reliant on the daily practices of community members, ensuring IFS for future generations requires consistent participation in traditional food practices at all levels of a community food system. The third principle of IFS is self-determination in relation to a community’s ability to identify and respond to their own needs, free from dependency on grocers and corporate food production. The final IFS pillar is policy, which aims to reconcile Indigenous food systems and values with mainstream laws, providing an opportunity for restorative policy reform of Indigenous Territories [24,26]. IFS strives to increase community control of Indigenous food systems as a platform towards self-determination, cultural reclamation [29] and restoring relationships [11]. IFS recognizes the ongoing impacts of colonization in communities and offers a human rights, land reform and self-determination approach because hunger is not the only impact a shift in food systems has for Indigenous communities [12,30]. Through generating an understanding of strategies that facilitate traditional food practices, IFS works to describe, rather than define, processes that support IK including hunting, gathering, growing, preparing and preserving traditional foods [26,30]. The complex nature of Indigenous food systems is part of the reason that IFS falls into its own category in comparison to other subsets of food sovereignty. IFS understands the strong connection between Indigenous Peoples and their traditional Territories and is ultimately reached when a community’s time-honoured interdependent relationships with all living and non-living things are nurtured and supported [26]. Researchers agree that community input is necessary to revitalize Indigenous food systems due to specific cultural needs [31].

IFS movements are working to strengthen Indigenous food systems across Canada that have been dismantled due to the impacts of environmental dispossession and the loss of traditional Territory. Specifically, over 80% of land in the province known as British Columbia is unceded territory. This land was not signed away by Indigenous communities and therefore, many settler cities and towns in this region exist on stolen Indigenous land [24,32]. Where treaties were negotiated, settler inhabitants and government forces have not honoured the original intentions of treaties to share the land between Indigenous Peoples and colonial settlers and have stripped Indigenous communities of their land rights [24]. For example, the Douglas Treaties, which encompass Indigenous communities all over Vancouver Island, initially intended to allow traditional food harvesting access on Indigenous Territories however, this was never employed in practice. Federal and provincial governing forces in this region prevented community members from leaving reserve communities (under the Pass system), dictated what communities could cultivate on-reserve, and what members could harvest off-reserve [33]. Through the limitation of on-reserve farming, the Canadian government restricted competition between Indigenous and non-Indigenous farmers, and created a favouring of settler farmers, further eroding the central relationship between Indigenous Peoples and their traditional foods [34]. The presence of heavy metals and other contaminants in many traditional food sources has created another barrier to engaging in traditional food practices for Indigenous communities [35]. Further, climate change has altered wildlife and aquatic migration routes, which communities rely on for hunting and fishing. Changes in temperature affect growing seasons, and community harvesting schedules. Ultimately, colonial settlement across Canada’s Pacific coast has been identified as a direct disruption to the intergenerational transference of food knowledge, resulting in decreased participation in traditional food practices and weakened Indigenous food systems [36]. Relationships between Indigenous Peoples and their food systems are complex; more research is needed to gain insight to better understand processes that can support IFS in Canada.

Due to the widespread impacts of colonization and processes of environmental dispossession on Indigenous Peoples’ health and food systems within Canada, there is a need for research to examine the influence of community collaboration in establishing sustained IFS [5,37]. Numerous IFS movements exist across Canada; in Manitoba, research conducted by Rudolph and McLachlan stressed the need for an evaluation of political contexts when striving to attain IFS [11]. In 2012, Rudolph used a knowledge exchange program to expand the use and consumption of traditional foods between southern and northern communities in Manitoba [38]. Participants in Cidro and Martens’ urban research project in Winnipeg identified the consumption and preparation of traditional foods as essential in reclaiming one’s cultural identity and as a mechanism for social connections [39]. While efforts to increase access to traditional foods are less common in the literature than efforts to identify barriers to traditional foods, participants from an urban study in Vancouver asserted that discussions around practical solutions rather than challenges were more effective in engaging community members [37]. The success of Kamal and Thompson’s country food program in northern Manitoba reiterated the positive impact of food-based action on Indigenous relations with the land and the impact of sharing traditional foods on sustainable livelihoods. Cidro et al. [18] and Receveur et al. [40] both identified the consumption of traditional foods as facilitating cultural values including sharing and responsibility. Projects on the Pacific coast have proven successful in providing space for transmission of IK between Elders and communities [36,37,41]. For example, the *Feasting for Change* program brought together Elders from across Vancouver Island to feast on traditional foods and share stories about both the loss and revitalization of these foods. Participation in this project made it apparent that participants had a desire for more opportunities to share and learn from one another [41]. Additionally, Elliot et al. [38] engaged in a collaborative project assessing obstacles and resolutions to accessing traditional foods in the city of Vancouver. Participants identified increased access to sea and land, food-related community programs, increased traditional food practices and valued cultural traditions as potential methods to increase access to traditional foods [38]. Through the establishment of a community-based food program, Kamal et al. [29] identified the engagement of youth and Elders in traditional land-based activities as central to the resurgence of cultural values and practices. The cultural implications of harvesting and preparing traditional foods have been identified as a remedy to multi-faceted health matters facing Indigenous Peoples living in British Columbia [29,42]. Previous IFS projects have been successful in bringing communities and people together to share knowledge and skills through meaningful experiences, however the literature does not address community needs for the sustainability of food sovereignty efforts and sharing of IK within communities, outside of research programs.

This article reflects a community-engaged research project designed to explore perspectives and experiences of Indigenous food sovereignty efforts on Vancouver Island. The overall purpose of the research was to better understand the community’s current IFS endeavours, and to generate a discussion of barriers and facilitators to furthering these efforts. This paper will examine the utilization of a community based participatory research (CBPR) theoretical framework in developing a research design that encourages discussion around facilitators and barriers First Nations community members face in pursuing IFS projects and planning.

## 2. Materials and Methods

Indigenous methodologies are fluid and dynamic processes that emphasize cyclical perspectives and alternative approaches towards research [42,43]. The primary focus of Indigenous methodologies is to ensure research is conducted in an ethical and respectful manner, these methods are stimulated by decolonization efforts and reconciliation aspirations [43]. Indigenous methodologies encourage researchers to approach their work as a learner, which is critical in creating sensitive, open-minded researchers that aim to abstain from misrepresentation, misinterpretation and exploitation [43,44]. Through the utilization of Etuaptmumk, or two-eyed seeing, this project employed Indigenous methodologies as a theoretical framework. Two-eyed seeing centralizes traditional beliefs, employs Western research methods and aims to engage comprehensive understandings that exist within IK systems [45]. Two-eyed seeing, “see(s) from one eye with the strengths of Indigenous ways of knowing, and see(s) from the other eye with the strengths of Western ways of knowing, and use(s) both of these eyes together” [45] (p. 335). The concept of two-eyed seeing was first introduced to research by Mi’kmaw Elder Albert Marshall; it is an important guiding principle that asserts that both Western and Indigenous ways of knowing are critical to Indigenous communities. This principle helps appreciate diverse perspectives of understanding the world, while enabling research teams to draw on what is helpful to grow existing knowledge [45].

CBPR provided a parallel theoretical framework to guide this project and ensured community input to guide the direction of research. CBPR develops research goals collaboratively with communities, based on a shared vision that helps remind all participating contributors of the potential benefits for the community [46,47,48,49]. In accordance with the Truth and Reconciliation Commission’s (TRC) Calls to Action, this research project was constructed with consultation from the community. The bi-directional nature of CBPR research ensures that research is conducted in a mutually beneficial manner, providing a supportive environment for knowledge sharing and co-creation [49]. In this project, one trainee and one senior researcher, worked in partnership with the community band council, leaders, and individuals involved in community food projects. CBPR has been identified as a catalyst for transformation because it defies the colonial tendencies of research to exclusively give power to the researchers. Previous work conducted using CBPR in Indigenous contexts in Canada has been successful in helping identify previously overlooked community needs [48].

Conducting research in line with the Tri-Council Policy Statement: Ethical Conduct for Research Involving Humans (TCPS2) principles, which promote research that is grounded in relationships and congruent with community priorities, is a requirement for all government funded research, including this project [50,51]. The TCPS2 articles also call for research that builds capacity in communities through hiring local community members, consulting with participants during data analysis to ensure cultural relevance and recognizing contribution to results [50]. This research project works towards the TRC’s Calls to Action numbers 18 and 19, which acknowledge the current state of Indigenous health as a result of government policies and work to establish measurable goals, in conjunction with Indigenous communities, to identify and close gaps in Indigenous health outcomes. Ethical considerations guided the direction of the research project and assured that the research was meaningful to the community. This project received approval from the University of Guelph Research Ethics Board and all participants gave their informed consent prior to participation in the project.

### 2.1. Research Setting

The traditional Territory of the First Nation community partner is situated on present day Vancouver Island, located on the Pacific coast of Canada. Historically, Coast Salish communities of this region had some of the highest population densities across North America due to the abundance of natural resources on the land, in the seas and rivers. Coastal communities on Vancouver Island were some of the first Indigenous communities to have extensive interaction with early settlers due to their geographical location. Colonial settlements in this region began in the 1840s, and many communities lost the majority of their Territory in May of 1850, under the Douglas Treaty. This treaty permitted settlers to hunt and fish on the lands and waters freely, effectively restricting local Nations’ ability to solely rely on their traditional food resources. The ongoing development of regional forestry, fishing and oil industries, among others, continues to further destroy local ecological habitats and sources of traditional foods from both the land and the waters. Engagement with this community was part of a larger team grant, based on over ten years of previous work and partnership with community leaders regarding renewable energies and Indigenous health.

### 2.2. Participants

Participants were elected by community leaders based on their involvement with the day-to-day food-related activities in the community to encourage a discussion of their experiences in their roles and the viability of different prospective food projects. Although discussions around having multiple perspectives included in the project were had, Elders in the community indicated that doing a piece solely with those actively involved in IFS pursuits at this time would be a preferred starting point for future work. Through purposive sampling, individuals involved in the community garden, traditional outings and harvesting events were identified [52]. Strong community relationships were crucial for recruitment as participants were identified through their roles in the community across two years of community engagement related to the research design and over four months of the first author (BP) working in the gardens and building relationships. Three community members, who maintain community food programs either through employment or volunteer work, participated in this research project. All three participants self-identified as women, and two as Elders from the community. Notably, the findings presented herein are limited as they only reflect women’s roles in IFS for this community. Once interest was confirmed, the research team followed up with a more formal invitation delivered directly to those wanting to participate.

### 2.3. Study Design

While the first author volunteered in the community garden and participated in harvesting and gathering events, informal conversations were held with participants to determine the most relevant research design. After months of morning talks in the gardens, and hikes with youth, specific areas of interest were identified and transformed to a sharing circle guide (Supplementary Material File S1), as a sharing circle was collectively chosen as the main method for gaining insight into current IFS efforts in the community. The utilization of a sharing circle combined with reflexive thematic analysis is a direct example of how this project employed two-eyed seeing [46], merging Indigenous and Western research approaches. By providing an open environment for participants to contribute their thoughts and knowledge with one another and with the first author, the sharing circle reflected the values of Indigenous methodologies, specifically two-eyed seeing, and CBPR practices [53]. Sharing circles allow increased engagement with participants, in comparison to interviews, because individuals are not restricted to answering questions but are also able to ask their own questions to further the discussion [48]. Traditionally, the knowledge holder determines what information they would like to share; by using sharing circles, participants are in control of their knowledge [53]. Further, the social aspect of sharing circles can contribute to a collective discussion of a topic and tends to more accurately mimic real life when participants are talking to each other, rather than directly to the researcher [54]. Conversational methods are significant in Indigenous methodologies because data is gathered orally, similar to traditional storytelling, consistent with an Indigenous paradigm [55]. A sharing circle guide was developed with participant input on conversational and open-ended questions to help promote storytelling and participant control over the conversation. The sharing circle occurred over a shared meal and lasted approximately three hours, the beginning of the sharing circle was researcher-driven, however over the course of our time together, participants began asking questions and the sharing circle became participant-driven.

The original intent of the project was to establish a community advisory group that would help determine community participation and recruitment, data collection methods, as well as specific research questions. In action, the research team consulted with individuals who had a stake in community food projects to determine what would be most helpful to support their work. By using a CBPR framework, the research team conducted research in a decolonizing and reconciliatory manner that empowered community members through the promotion of an ongoing, positive relationship with traditional foods. A qualitative research approach enabled an in-depth exploration of concepts and permitted room for IK to emerge as qualitative methods do not assume one correct understanding of reality [50]. CBPR was actualized in this project through the development of relationships over two years between the research team (BP and HN) and tweaking of project aims happened on an ongoing basis once BP relocated to the community, due to daily conversations with Community leaders and Elders working in the garden.

### 2.4. Data Analysis

Braun and Clarke’s framework for reflexive thematic analysis guided the analytic process. Reflexive thematic analysis embraces the unique subjective skills a researcher brings to the project and enables organic identification of themes [54,56]. As such, it is critical to acknowledge the assumptions one brings to qualitative research as they impact the interpretation of data and production of findings. As a non-Indigenous researcher from Canada, the primary author took steps to familiarize herself with the data and the context in which it was collected through the building of relationships and time spent working alongside community members in the gardens over two years. Understandings of data were discussed extensively with community members that participated in the sharing circle prior to initiating analysis.

The sharing circle was audio recorded in the field and electronically downloaded and transcribed verbatim by the research team. Inductive themes, grounded in the data, were coded line by line and without a codebook to enable engaged interpretation of data. Once all transcripts had been coded, the data was re-visited, and similar codes were aggregated for iterative thematic development [54,56]. NVivo 12 software (QSR International Pty Ltd., Burlington, MA, USA, Version 12.6.1) was utilized to complete reflexive thematic analysis of the sharing circle. Once themes were identified from the sharing circle, a general short report was prepared for the community and shared with participants. At this time, feedback on the initial analysis of the content was generated by community members and after consensus on changes was reached with all participants, revisions to analysis and conceptual models were made accordingly.

## 3. Results

**Figure 1:**
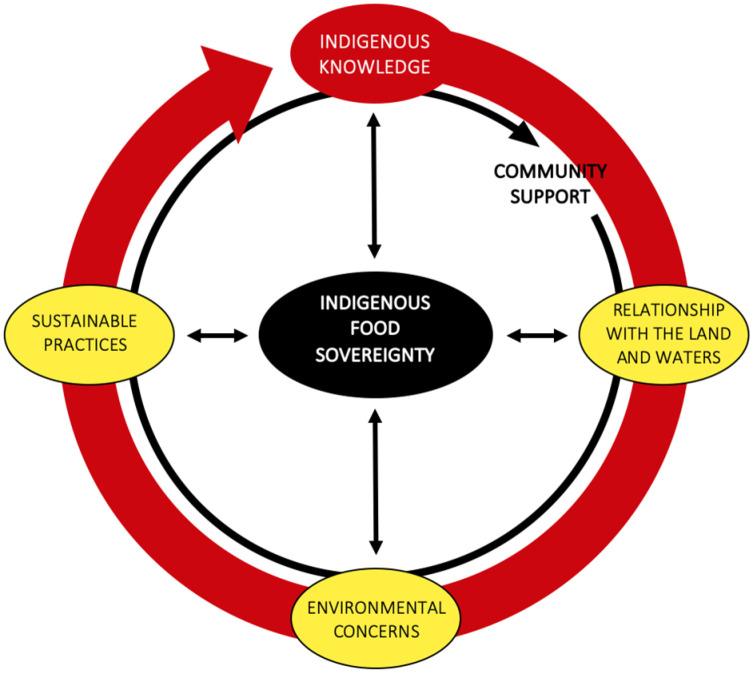
Thematic Model of Community Food Sovereignty Experience and Practice.

The interconnected nature of the thematic results (Figure 1) reflects a holistic understanding of Indigenous food systems. All findings impact the community’s IFS efforts, either facilitating or creating a barrier to food projects. Participants’ enthusiasm for the growing, harvesting and consuming of traditional foods was evident through their excitement when discussing IFS efforts in the community. Topics of climate change and environmental concerns constituted a large portion of discussions, even though none of the sharing circle questions focused on this theme. The central theme of IFS, as placed at the heart of Figure 1, encompasses participants’ memories of harvesting and gathering foods as children, the ongoing work at the community garden, the workshops focused on traditional methods and the weekly community meals. The themes of IK and community support are presented in a cyclic shape to represent the continual impact of these forces on the community’s actions and values; these themes were the motivators for why participants dedicated their time to community-level IFS. Community support provided a strong foundation for all food projects and cultural revitalization efforts discussed by participants. A strong desire to pass on IK to future generations was evident throughout the discussion of various community projects. IK informed the participants’ beliefs and understandings of the facilitators and barriers that arose from the sharing circle, including environmental concerns, relationship with the land and waters, and sustainable practices.

### 3.1. Indigenous Food Sovereignty

Discussions throughout the sharing circle focused on IFS efforts in the community, as well as the barriers and facilitators to these projects. The outer rings in black and red, as seen in Figure 1, each directly influence the community’s understanding of their own food sovereignty and inform the values that underpin all of their current projects. Participants discussed various community activities that support IFS efforts, including the community garden; food grown at the garden is used for weekly community luncheons, culture night dinners, an Elder meals-on-wheels program and when in excess, distributed amongst community members. The community garden is valued as a supplier of food for the Nation, rather than a revenue stream, and focuses on growing and harvesting produce for the wider community. Additionally, medicinal teas and herbs are grown at the community garden, where they are thoroughly dried and jarred for distribution in the community and for use at workshops. Participants also discussed workshops that are held at the band hall including preservation and smoking classes; developing these traditional skills help community members reinforce their IK and teaches them how to utilize food sources from their own backyards, rather than solely relying on local grocers. Participants explained that when they take members out to harvest traditional foods, they duplicate traditional methods that their ancestors followed to spread IK amongst youth and the community. One of the participants stressed the importance of teaching children traditional practices to prevent the loss of IK:

*“When we go out, we harvest the berries as we did, teaching the children that is really important because now they’re gonna teach their children. A lot of that knowledge is gonna get forgotten ‘cause nobody would know how to do it, or where to go, what to do with it when they get it.”*

Participants also discussed a group of members within the community that harvest and distribute fish amongst the households within the community each year. When directly asked about what is needed to sustain the IFS efforts within community, participants responded unanimously that participation from the wider community is what sustains the food projects.

### 3.2. Indigenous Knowledge (IK)

Through growing food at the community garden, leading spiritual prayers, organizing food gatherings or coordinating harvests; each of the participants are a rich source of tradition and culture within the community. Participants heavily value the ways in which their ancestors lived. On multiple occasions, participants expressed that the ways their ancestors prepared or harvested food and “lived simply” as the ‘right’ way to do things. When discussing the fulfilling aspects of organizing traditional workshops and outings, one participant exclaimed: “The most rewarding and fulfilling I think with the food is not only watching people enjoy what it is, we usually prepare it the way our ancestor did so that they enjoy it the way they’re supposed to”. The sharing and preservation of IK was extremely important to the participants to ensure that the knowledge stays within the community for generations to come as illustrated by discussions of intergenerational knowledge transfer to youth. One of the participants shared language cards developed specifically for youth outings, so that youth can learn plants’ identification and traditional names simultaneously. IK and associated community values inform the themes of environmental concern, sustainable practices and relationship with the land and waters. Participants expressed that their ancestors engaged in practices that supports each of these themes.

### 3.3. Community Support

Community support was woven throughout the discussions of IFS with community members. Specifically, when looking at IFS, participants identified that while the community garden is supported by annual grant applications, when grants do not cover budgetary needs, the Chief and Council will allocate money from the community budget to support the garden. With regards to the relationship with the land and waters, participants stated that they encourage participation from all community members, regardless of age or ability. One participant shared that they actively alter outings to make it accessible for all members: “Whether they are in a wheelchair or they can walk slowly or they have some kind of ailment that can’t make them go very quickly, it doesn’t matter because you go at their pace”. Participants identified the need to get back on the land and waters, away from modern technology, that can cause community members to remain in the confines of their homes. Recognizing the wider need for individuals to be active and get outside helps support the community and encourages a fruitful relationship with the land and waters. The participant responsible for the planning of traditional outings and workshops articulated the following:

*“Our whole method to our madness for gathering is to get people out of the house, away from gadgets and TVs and for our Elders to move around and… You make sure that they get it done. I just think it’s wrong that people sit in their houses with all that stuffiness.”*

Further, encouraging participation from those that are hesitant yields great results according to participants. Elders feel useful when they are encouraged to share their IK and they are able to enjoy the world as they once did when they were young, and their Elders were teaching them the same practices they are sharing with the current youth. While the participants discussed community and support, especially when expressing motives for growing food, teaching prayers and organizing workshops, they also identified the need for reciprocal support. When directly asked what is needed to ensure the future of IFS in the community, one participant replied: “Participation, participation, participation”. Community engagement is what fuels both ends of the IFS efforts, whether through volunteering at the community garden, participating in a canning workshop, or going on a berry harvest hike, participants identified this as the number one necessity for continuing their work in the community.

### 3.4. Facilitators and Barriers

#### 3.4.1. Environmental Concern

Participant concern for their ecological environment was strong throughout the sharing circle, as evidenced by the numerous occasions concerns such as forestry, pollution and climate change were discussed both as a direct response to prompts and as an indirect or related response. While environmental concern itself, was discussed as a facilitator and motivator to IFS, the items of concern, such as climate change, are creating barriers to successful IFS. Community members provided clear examples as to how the changing climate is negatively affecting their ability to continue traditional practices, sustain IFS efforts and spread knowledge amongst membership. Specifically, participants have noticed that traditional food sources, both in the garden and in the wild, are not as successful as they were even 5 years ago. As articulated by one of the participants who has been gathering traditional foods since childhood:

*“Especially the native plants because they’ll flower and that’s it, they won’t bear fruit, or whatever they’re supposed to grow, a lot of times when we go out to gather the fruit, they’re airy and they don’t have enough of the moisture that they’re supposed to get, they’ll grow to a certain size and stop and become little balls of air, not fruit. Even the seeds won’t grow, and that means a lot because the seeds are what make more, right? When they fall off the plant. And that’s concerning because it’ll just go away and not replenish itself.”*

Participants’ IK and beliefs, as well as a strong understanding of the interconnected nature of their surrounding environment, played a role in their comprehension of the current state of the environment. In discussing the cyclic nature of the environment and the impact of disrupting those cycles, one participant stated:

*“Yeah, the trees are what cleans the air and keeps our climate, it’s a system, everybody knows that from going to grade school. If the system is stopped midway, we’re teetering, but they keep taking all the trees so that the air can’t do what it’s supposed to do, filter through the trees.”*

Development in the nearby city, forestry and commercialization has negatively impacted ecosystem health of the community’s traditional Territories and participants identified these forces as a catalyst for eliminating traditional food sources. Two of the largest berry sources for the community have been destroyed; one due to the building of a coffee shop and the other due to forestry demands. One participant reflected upon the importance of the second berry patch throughout her life:

*“There’s nothing there anymore, because they took it away. That’s forestry, that’s taking the trees, that’s wrecking it- oh my god. There’s nothing out there anymore, it is really sad. It’s like wow, we went there for years and years and years, even when we were kids we went out there and now it’s gone.”*

The impact of plastic use was also an environmental concern expressed by community members during the sharing circle, in terms of ocean pollution and wildlife habitat destruction. Participants called for an immediate end to the use of plastics, citing them as unnecessary waste and a threat to wildlife and ecosystem wellbeing. Community members also shared the desire to be able to conserve their ecosystem on multiple occasions but expressed uncertainty about how to ensure preservation of their traditional food sources. Specifically, one participant asserted concern for the stability of all food systems:

*“You know there’s going to be one day when all those vegetables aren’t going to be in the grocery store because the greenhouse effect, it’s all changing, it’s changing fast and a lot of people don’t know how to correct it. Yet it’s everybody’s fault for wrecking it because they shouldn’t have been allowed to do what they did to wreck it in the first place.”*

#### 3.4.2. Relationship with the Land and Waters

Through a discussion of traditional practices, it became apparent that a strong relationship with the land and waters was central to many of the participants’ IK. The participants expressed the impact of having Elders remind youth of the cultural value traditional Territories continue to hold through the teaching of harvesting methods, as invaluable. The organizer of community outings highlighted the value of sharing stories in establishing a relationship with the land and waters for youth: “Listening to their stories of when they were youth and then our youth understand why we are doing it. The importance of our lands, our beaches, we need to live off the land. It’s so important and it’s still out there”.

When discussing the growing of traditional foods as a result of a relationship with the land and waters, participants expressed this relationship is central to their identity. The extraordinary concern for the environment expressed by participants reinforces this sentiment. When discussing the current state of the ecological environment, participants identified that preservation of the land and waters is required for the health of future generations. Further, the community’s strong relationship with the land and waters as well as their understanding of the interconnectedness of nature, was identified as central to the community’s spirituality. In the community, prayer is used as a way to start ceremonies and events by acknowledging members’ presence on traditional Territories and highlighting the importance of gratitude and respect for all living things. Most importantly, participants identified that true respect of Mother Earth and nature would not lead to the current climate crisis: “That’s where people need to be, is respect. If you respect Mother Earth, you wouldn’t destroy her”.

#### 3.4.3. Sustainable Practices

Many of the traditional practices discussed by community members reflected a sustainable way of living, respecting Mother Earth and prioritizing the health of the environment. A strong desire to return to a simpler time where priorities reflected ancestral values was apparent throughout the discussion. Participants discussed the importance of the seven generations law, from the great laws of the Iroquois confederacy, in the community, which considers the impact of today’s decisions on tomorrow’s generations. As described by one of the participants, all efforts to consider this impact are made by community members: “We always think ahead, sometimes it doesn’t pan out, but most of the time it does, in whatever we do, just gotta keep pushing it, pushing it on our community to be sustainable”. Another example of sustainable traditional practices given by the participants was the collecting of rainwater in barrels, a regular practice for community members even one to two generations ago. All of the participants’ grandparents used rainwater to do laundry; this practice is a direct example of the traditional value placed on water as a precious resource not to be wasted. When reflecting on traditional practices, in comparison to development practices of the Western world, participants reiterated that they were taught to only ever take what is needed from their environment in contrast to colonial consumerism values. This mindset in and of itself is a core principle that reflects the sustainable nature of the community’s traditional way of life. When discussing harvesting practices of the forestry industry on Vancouver Island, one of the participants asserted: “We never did that; our people never did that. They would only take whatever they needed at the time”.

## 4. Discussion

While several researchers have documented and analysed food sovereignty movements [12,31,57], many projects working with Indigenous communities tend to use a food security lens [21,25,37,58] and IFS projects [18,29,30,59,60] are less frequently investigated. This project is unique in that it utilized CBPR to guide the direction of the project, ultimately exploring local understandings of IFS; the resulting discussions from the sharing circle were effective in identifying facilitators and barriers experienced by women invested in the local food system and sovereignty movements. The results from the sharing circle focused on the impact of the changing environment on restricting the community’s ability to harvest traditional foods and sustain Indigenous food systems. Bringing to light the concerns that participants have for the future of food in the community will enable us to continue working with the community to generate potential solutions. Environmental concerns, as a result of settler-induced climate change, are closely interwoven with the ability of marginalized communities to pursue food sovereignty efforts and will become an increasingly prominent field of study as the impacts of climate change intensify, as is the case for Inuit communities in Canada who are at the forefront of these changes [61]. The limiting impacts of climate change on IFS in this community are illustrated through decreased access and availability of traditional foods, as well as altered migratory patterns and growing seasons.

Through the exploration of gardening as a mechanism to support Indigenous health in the context of climate change, Timler and Sandy [58] discuss the necessity of a shift towards a relationship of reciprocity with food and the land in research to ensure adequate climate negotiations and policy agendas that address the health inequities experienced by Indigenous Peoples. The notion of reciprocity was central to all themes identified from the sharing centre in relation to respect for Mother Earth. This concept is further supported by Kimmerer’s [62] discussion of reciprocity amongst all living things, where the Indigenous way of life is represented as duties and responsibilities to living things that sustain human life. Kimmerer ascertains that while humans are not in control of the natural world, they are responsible for respecting all living things through harmonious living patterns that honour the value of the land [63]. In the Canadian context, the colonial history of the country is often overlooked amidst IFS conversations. This is problematic for Indigenous communities because, as identified during the sharing circle and previous research, modern consumerism practices support an economy that preys on Mother Earth and disrupts the essential and reciprocal relationship between humans and the natural world [42,59]. During the sharing circle, participants identified the astronomical impact human development has had on their ecological environment and the tendency of people to ignore their destructive habits. Despite the human-inflected abuse of our planet, Mother Earth continues to support the human race [42]. As articulated in the literature and discussed by participants, the Indigenous understanding of the concept of sustainability is intrinsically tied to the sharing of IK and the teaching of cultural practices across generations [59]. The connection between the thematic findings of environmental concern, sustainable practices and a relationship with the land and waters is rooted in mindfulness for the environment and respect for the physical world around us. This understanding reflects a deep desire to conserve the natural world that aligns with the reciprocal nature of Indigenous ways of life [59]. The importance of a strong relationship with their traditional Territories informed all that the participants discussed. In establishing a collective gardening place to promote wellbeing amongst imprisoned Indigenous men in British Columbia, participants from Timler and Brown’s [64] project identified participation in gardening as a mechanism for personal healing and strengthening of relationships, established and supported through gifting of produce from the garden. The importance of the relationship with the land for Indigenous communities has been well documented as many works have identified the health of land to be almost synonymous with the health of Indigenous communities [16,17,59,65].

The findings from this work indicate that IK, sustainable practices, relationship with the land and waters, and community support facilitate IFS, while environmental concerns create a barrier. These results are similar to Robin’s [60] proposed IFS foundation, which includes connection to land and water, cultural identity, history, and relationships. Both projects presented findings (Figure 1) in a cyclical diagram, representative of the process of IFS, and similar to the Indigenous Medicine wheel [60,66]. While Robin’s [60] work established a general foundation of IFS through an exploration of various community projects in Western Canada, our project is unique in presenting an in-depth experience of one community and highlights both the facilitators and barriers to maintaining IFS projects in this context. Defining the meaning of IFS was not the goal for either of these projects, there will never be a singular definition for this process, as Morrison illustrates: “While there is no universal definition of food sovereignty that reflects all of the realities of the myriad of Indigenous communities around the world, the underlying principles of Indigenous food sovereignty are based on our responsibilities to uphold our distinct cultures and relationships to the land and food systems” [23] (p. 97). Exploring and documenting processes at the community level is effective in providing avenues for future local projects and in generating considerations for communities wanting to develop projects [60].

When directly asked about what is needed to further community-level IFS efforts, participants responded unanimously that engagement from the wider community is what sustains their efforts. An important aspect of IFS, as noted by Coté [59] is acknowledging the efforts being made by communities to fortify their local food systems and support traditional relationships with the land and waters. Returning to traditional food practices through the sharing of IK and the utilization of Elder wisdom in planning food projects supports community values and furthers IFS by empowering members to participate in tradition that often times aid in revitalizing cultural identity [7]. Similarly, the inability to participate in traditional practices, such as hunting and gathering, prevents a strong relationship between individuals and the natural world, jeopardizing Indigenous cultures [59]. Participants in this project highlighted the necessity to modify food outings and gatherings in order to accommodate community members of all ages and abilities to create inclusive environments. While Timler and Sandy’s work [58] primarily functions through a food security lens, the importance of intergenerational education as a facilitator of future food sovereignty efforts and an alternative to the colonial present, was similarly identified amongst their participants, as in our project. Timler and Sandy’s project concludes with a call to widen the conversations around IFS in Western Canada to gain a more holistic understanding of community experiences [58], this project answers that call.

While mechanisms to investigate IFS vary due to the diverse needs across the differing cultures and environments in communities, this project demonstrated that exploring community perspectives can help gain insight into community values and priorities with regards to sovereignty efforts. This project did not examine political impacts, treaty negotiations or conservation efforts as related to food sovereignty, all of which are critical components to ensuring effectiveness of IFS efforts. The local food programs discussed cannot be considered in isolation from impacts of resource extraction, overhunting, industrialization and community governance, to name a few. The aforementioned loss of social, economic and political independence due to forced assimilation and colonization has had a tremendous impact on the modern Indigenous food systems in Canada [11]. Therefore, it is impossible to fully understand sovereignty efforts and effect sustained change without acknowledging all components of the complex issues faced by Indigenous Peoples in Canada. This project is further limited due to the small number of female participants and is therefore not representative of the wider community. These findings cannot yet be generalized to this community or to other Indigenous communities in the area, but future work will help develop the findings presented here. This project is the first step of a larger research piece that will build on what was established during this initial data collection. Potentially, the three participants from this stage of the research will become the official advisory committee and will help guide the subsequent interviews and community collaboration. The next step of this research will be to widen the scope of perspective by including community members not directly involved with food efforts to generate insights on how food sovereignty efforts impact the lives of the community as a whole.

## 5. Conclusions

The emerging themes from this project, IK, environmental concerns, sustainable practices, relationships with the land and waters and community support, provide a framework for understanding facilitators and barriers to community-level IFS. For the community partner, this project provides the basis for future exploration of ways to overcome identified barriers and enhance facilitators. The establishment of sustained funding for the community garden and increased participation in food project infrastructure would alleviate some of the barriers to IFS discussed by participants. Additionally, further discussion of specific environmental concerns in the community would be beneficial in creating a strategic plan to mitigate impacts of climate change moving forward. Finally, pursuing conservation efforts to protect local food sources and strategies to alleviate the impact of invasive species on the land and in the water will help protect food sources that are central to the community’s cultural identity. The next steps of this research project will be a consultation with the wider community, including interviews to further explore the idea of IFS and to generate a holistic understanding of the community’s vision for the future of food.

These results will also help inform literature around Indigenous perceptions and perspectives of food sovereignty. Documenting various positive aspects of IFS is necessary to shift the narrative of Indigenous wellbeing within Canada. Many projects still highlight the negative health disparities and do not provide a holistic, encouraging story for Indigenous communities. Prioritizing the Indigenous voice in documenting IFS efforts is essential because Indigenous Peoples are the everyday participants in their lives and ultimately, will benefit from successful implementation of food programs that support sovereignty. Further, promoting discussions of community perspectives, rather than analysing community situations, is crucial when working to promote sovereignty; despite efforts to utilize Indigenous methodologies, research analysis often remains an outside perspective. The strengthening of food sovereignty movements is critical to the overall wellbeing of Indigenous communities within Canada, due to the prioritization of consumption of traditional foods and land-based learning present in IFS projects. The findings from this project describe the continued importance of traditional foods and more generally, traditional lands and waters in sustaining the health of communities. Finally, this work supports the previously identified need for a “swift paradigmatic shift” to an understanding of the relationship between human wellbeing and the environment [67]. There is still much work to be done in the realm of IFS and future projects should aim to describe community understandings of food sovereignty, as well as the facilitators and barriers to achieving local goals for food systems, so that meaningful work can take place and avenues to support community efforts can be discovered. Traditional practices and Indigenous food systems are sustainable in nature, increasing accessibility to knowledge and confidence around traditions will encourage community members to initiate and partake in these practices more frequently to ensure the longevity of community-level IFS.

## Data Availability

The dataset analysed during the current study are not publicly available due to confidentiality concerns.

## Supplementary Materials

The following supporting information can be downloaded at: https://www.mdpi.com/article/10.3390/ijerph20054627/s1, File S1: Sharing Circle Guide.
