# Supplementary material for: “We Need to Live off the Land”: An Exploration and Conceptualization of Community-Based Indigenous Food Sovereignty Experiences and Practices
Source: Int J Environ Res Public Health. 2023 Mar 6;20(5):4627. doi: 10.3390/ijerph20054627 (PMC10002046; doi:10.3390/ijerph20054627)
Supplement: Supplementary file 1 [file ijerph-20-04627-s001.zip › ijerph-2224295-supplementary.pdf]

Sharing Circle Guide

1. Start with introductions & short description of everyone’s role in the community.
2. What is your favourite memory of food?
3. What are some of the traditional food practices of T’Sou-ke Nation?
4. What is most rewarding or fulfilling about your work with food in the community?
5. What is the most frustrating part about your work with food? Where would you like to see improvements?
6. What role can the wider community play in furthering the success of food sovereignty efforts?
7. T’Sou-ke has been extremely successful in implementing solar power throughout its community; what has been the biggest impact of the shift to solar?
8. Considering food and solar as products of a relationship with the land, how important is that relationship to you?
9. What concerns do you have for the health of the environment?
10. In an ideal world what would you envision for the future of food and energy sovereignty in T’Sou-ke Nation?
